# Why are tumour blood vessels abnormal and why is it important to know?

**DOI:** 10.1038/sj.bjc.6604929

**Published:** 2009-02-24

**Authors:** J A Nagy, S-H Chang, A M Dvorak, H F Dvorak

**Affiliations:** 1 Center for Vascular Biology Research, Beth Israel Deaconess Medical Center and Harvard Medical School, Baston, Massachusetts, USA; 2 Department of Pathology, Beth Israel Deaconess Medical Center and Harvard Medical School, Boston, Massachusetts, USA

**Keywords:** angiogenesis, arteriogenesis, venogenesis, tumour blood vessels, VEGF-A

## Abstract

Tumour blood vessels differ from their normal counterparts for reasons that have received little attention. We report here that they are of at least six distinct types, we describe how each forms, and, looking forward, encourage the targeting of tumour vessel subsets that have lost their vascular endothelial growth factor-A (VEGF-A) dependency and so are likely unresponsive to anti-VEGF-A therapies.

It has been known for more than a century that tumours have their own blood supply and that, for the better part of that time, the tumour vasculature is highly abnormal. At one time it was thought that the tumour vasculature was actually superior to that of normal tissues; this misconception arose because tumour vessels are often of large size and were, therefore, more conspicuous than the smaller, more numerous and functionally more effective blood vessels of normal tissues. By the early 1970s, however, it was clear that the overall blood flow tended to be significantly lower in tumours than in normal tissues, and that tumours needed to induce a vascular supply if they were to grow beyond minimal size. It was also suspected that tumours induced their neovasculature by secreting angiogenic factors, but the identity of these factors was just beginning to be investigated. In the years that followed, much has been learned about the molecular basis of angiogenesis and particularly about the central importance of one angiogenic factor, that is vascular permeability factor/vascular endothelial growth factor (VPF/VEGF, VEGF-A). Subsequent work has elucidated the steps and mechanisms by which VEGF-A induces the formation of tumour blood vessels. Furthermore, recent successes with agents that block VEGF-A or its receptors have supported Folkman's (1971) thesis that anti-angiogenesis can be a useful supplement to traditional tumour therapy. However, current drugs that interfere with VEGF-A function have not proved to be a cure-all (Jain *et al*, 2006). Their limited effectiveness, serious side effects and high cost have prompted a rethinking of some of the basic issues involved in tumour angiogenesis.

This minireview addresses several specific questions that are relevant to these basic issues: what are tumour vessels and in what respects do they differ from normal blood vessels? How do tumour vessels form? Why are they abnormal? And, finally, why might an understanding of tumour blood vessel diversity be important clinically? Hopefully, addressing these questions will clarify some of the mechanisms by which tumours induce their abnormal blood vessels and lead to the development of therapies that more effectively target them.

## What are tumour blood vessels and how do they differ from normal blood vessels?

**Figure 1:**
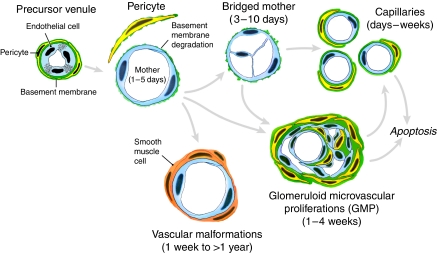
Schematic diagram of the angiogenic response induced by Ad-VEGF-A^164^. Modified from Pettersson *et al* (2000).

Tumour blood vessels have often been regarded as if they were a single entity, ‘angiogenic blood vessels’. However, it has long been known that tumour blood vessels are heterogeneous with regard to organisation, function and structure (Warren, 1979). Whereas the normal vasculature is arranged in a hierarchy of evenly spaced, well-differentiated arteries, arterioles, capillaries, venules and veins, the tumour vasculature is unevenly distributed and chaotic. Tumour vessels often exhibit a serpentine course, branch irregularly and form arterio-venous shunts (Warren, 1979). Blood flow through tumours does not follow a constant, unidirectional path. Not all open vessels are perfused continuously, and, over a few minutes, blood flow may follow different paths and even proceed in alternating directions through the same vessel (Chaplin *et al*, 1987). Tumour blood vessels are more abundant at the tumour–host interface than in central regions. Also, vascular density tends to decrease as tumours grow, leading to zones of ischaemia and ultimately necrosis as tumours ‘outgrow their blood supply’ (Peterson, 1991). Finally, tumour blood vessels are structurally abnormal. On the basis of their anatomic and functional properties, we have recently classified tumour blood vessels into at least six distinct types. These differ from each other, and, with the possible exception of tumour capillaries, differ from the vessels found in normal tissues (Table 1, Figure 1).

## How do tumour blood vessels form?

**Figure 2:**
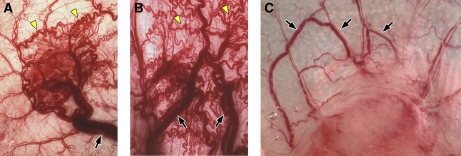
Vascular patterns induced by Ad-VEGF-A^164^ (**A**, **B**) and by MOT, a mouse ovarian tumour (**C**). (**A**, **B**) FA and DV (black arrows) at 27 and 59 days after s.c. injection of 10^8^ PFU of Ad-VEGF-A^164^. Most angiogenic vessels except for VMs (yellow arrows) have resolved. (**C**) Mouse ovarian tumour 10 days after s.c. implantation. Black arrows indicate some FA and DV; T, tumour.

Tumour angiogenesis is a relatively crude process that results from the unbalanced secretion of a small subset of cytokines, particularly VEGF-A (Dvorak, 2003, 2007; Nagy *et al*, 2007). Recent studies with adenoviral vectors expressing VEGF-A^164^ (Ad-VEGF-A^164^) have contributed importantly to our understanding of the mechanisms by which tumours generate new blood vessels. When injected into the tissues of immunodeficient mice, Ad-VEGF-A^164^ induces the formation of each of the different types of tumour blood vessels listed in Table 1 and Figure 1. Similar to tumour vessels, which show only limited tissue specificity, the surrogate blood vessels induced by Ad-VEGF-A^164^ are largely independent of the tissue in which they arise; similar vessel types form with similar kinetics in a wide variety of normal mouse and rat tissues, including skin, subcutis, fat, skeletal and heart muscle, and brain (Pettersson *et al*, 2000; Stiver *et al*, 2004). The first type of new blood vessel to form in response to Ad-VEGF-A^164^ has a characteristic morphology and has been given the name ‘mother vessel’ (MV) (Pettersson *et al*, 2000). However, similar MV-like blood vessels are also induced by polymers containing VEGF-A^164^ or basic fibroblast growth factor (Cao *et al*, 1998) and by tumours expressing VEGF-A or basic fibroblast growth factor and platelet-derived growth factor-BB (Paku and Paweletz, 1991; Bjorndahl *et al*, 2005; Nagy *et al*, 2007; Nissen *et al*, 2007). The other types of angiogenic vessels evolve from MVs and thus may be properly regarded as ‘daughter’ vessels (Figure 1) (Pettersson *et al*, 2000; Dvorak, 2003; Nagy *et al*, 2007). In addition to angiogenesis, tumours and Ad-VEGF-A^164^ induce abnormal arteriogenesis and venogenesis, thereby generating large vessels that feed and drain the angiogenic vascular bed (Figure 2). Here we summarise what is known about each of these vessel types and how they form.

## Mother vessels

Mother vessels are highly permeable sinusoids that begin to develop from pre-existing venules and, to a lesser extent, from capillaries within hours of injection of tumour cells or Ad-VEGF-A^164^ into mouse tissues. Mother vessel formation involves a three-step process of basement membrane degradation, pericyte detachment and extensive enlargement. Basement membrane degradation is an essential early step, because basement membranes are non-compliant (non-elastic) structures that do not allow microvessels to expand their cross-sectional area by more than ∼30% (Swayne *et al*, 1989), that is, far less than the three- to five-fold enlargement characteristic of MVs. We have recently shown that venular basement membrane degradation is mediated by an increased expression of pericyte cathepsins, coupled with a decreased expression of cysteine protease inhibitors by both pericytes and endothelial cells (unpublished data). This upsetting of the local cathepsin–cysteine protease inhibitor balance leads to basement membrane degradation and detachment of pericytes, thus removing the constraints that normally limit microvascular size.

Rapid vascular enlargement also requires an increase in plasma membrane. This is provided, at least in part, by vesiculo-vacuolar organelles, which are clusters of hundreds of interconnected vesicles and vacuoles contained within the cytoplasm of normal venular endothelial cells. They have an important role in the transport of macromolecules across venules in the vascular hyperpermeability induced by VEGF-A, histamine, etc. (Dvorak *et al*, 1996; Nagy *et al*, 2008). Vesiculo-vacuolar organelles also provide abundant intracellular membrane stores that amount to more than twice that of the endothelial cell plasma membrane. As MVs develop, venular endothelial cells become thin and vesiculo-vacuolar organelles decrease in number and complexity because they contribute their membranes to the greatly expanded plasma membrane.

## Daughter vessels: capillaries, glomeruloid microvascular proliferations and vascular malformations

Mother vessels form and are maintained only as long as high concentrations of VEGF-A are present. Their thin walls, sluggish blood flow and a lack of pericyte and basement membrane support make them susceptible to thrombosis or collapse. Thus, they are transitional structures, present only for a limited time, and evolve into one or another type of stable daughter vessel (Table 1, Figure 1).

## Capillaries

One mechanism by which MVs evolve into capillaries involves intraluminal bridging, a process that was originally discovered in tumour vessels (Nagy *et al*, 1995), but which has since been found to occur in the angiogenesis induced by Ad-VEGF-A^164^ (Pettersson *et al*, 2000) and healing wounds (Ren *et al*, 2002), and after chronic vascular dilatation (Egginton *et al*, 2001). Endothelial cells extend cytoplasmic processes into and across MV lumens, forming transluminal bridges that divide blood flow into multiple smaller-sized channels. Subsequently, these smaller channels separate from each other to form individual, capillary-sized vessels that are, as far as is known, normal in structure and are not hyperpermeable.

## GMPs

Glomeruloid microvascular proliferations (GMPs) are found not only in a wide variety of VEGF-A-expressing human tumours, particularly glioblastoma multiforme, but also in cancers of the stomach and breast, where they are associated with an unfavourable prognosis (Pettersson *et al*, 2000; Sundberg *et al*, 2001; Straume *et al*, 2002). Glomeruloid microvascular proliferations are hyperpermeable, but, because they are poorly perfused, account for relatively little plasma extravasation. They develop from the proliferation of large, poorly differentiated CD31- and VEGFR-2-positive cells, which divide MV lumens into much smaller channels (Pettersson *et al*, 2000; Sundberg *et al*, 2001). Glomeruloid microvascular proliferations subsequently acquire pericytes and deposit extensive layers of abnormal basement membrane. Similar to MVs, GMPs require exogenous VEGF-A^164^ for continued formation and maintenance.

## Vascular malformations

Other MVs evolve into vascular malformation (VM) by retaining their large size and acquiring a smooth muscle-cell coating. Vascular malformations are readily distinguished from normal arteries and veins by their inappropriately large size (for their location) and by their thinner and often asymmetric muscular coat. Vessels of this description closely resemble the non-malignant VMs that occur, for example, in the skin, brain, etc. (McKee, 1996). Vascular malformations are not permeable to plasma proteins. Moreover, unlike MVs and GMPs, VMs persist indefinitely in a low VEGF-A environment, although it is quite possible that their lining endothelium is maintained by VEGF-A secreted by their smooth muscle-cell coat.

## Feeder arteries and draining veins

In addition to inducing angiogenesis, tumours and Ad-VEGF-A^164^ also stimulate abnormal arteriogenesis and venogenesis, leading to the formation of the large, often tortuous blood vessels that supply and drain the tumour microvasculature (Figure 2). These vessels have been little investigated. They are generally larger than VMs and acquire structural properties that are intermediate between arteries and veins. Similar to VMs, they persist indefinitely in a low VEGF-A environment.

## Why are tumour vessels abnormal?

Tumours initiate new blood vessel formation by the local, unbalanced overexpression of VEGF-A. As was noted more than 20 years ago, tumour angiogenesis shares many analogies with wound healing (Dvorak, 1986). Thus, at one level, tumours can be regarded as ‘wounds that do not heal’. Among the similarities are high levels of local VEGF-A expression, generation of characteristic MVs and generation of capillaries that result from intraluminal bridging of MVs. Of course, there are also some notable differences. One is that, as wounds heal, VEGF-A overexpression ceases. This is because the ischaemic stimulus upregulating the VEGF-A expression in healing wounds diminishes as new blood vessels form and restore local oxygen concentrations. By contrast, tumours continue to overexpress VEGF-A unabated, because, in tumour cells, the VEGF-A expression is driven not only by ischaemia but also by oncogenes, loss of tumour suppressor genes, hormones, etc. (Dvorak, 2003).

However, why does VEGF-A generate the abnormal blood vessels characteristic of tumours and healing wounds? At least three factors appear to be important: the total amount of VEGF-A present locally, the different VEGF-A isoforms that are expressed and the distribution of VEGF-A relative to target blood vessels. In tumours, healing wounds, and in the Ad-VEGF-A^164^ model, normal blood microvessels are bathed in high concentrations of VEGF-A, and the concentrations present locally seem to be of critical importance in determining the character of the new blood vessels that form. Intramuscular injection of cloned myoblasts that secrete low amounts of VEGF-A is reported to generate non-leaky, relatively normal capillaries, whereas clones secreting larger amounts of VEGF-A induce typical MVs (Ozawa *et al*, 2004). Also, in a model of retinal prematurity, an increased VEGF-A caused endothelial cleavage planes to orient laterally, rather than longitudinally, leading to the formation of enlarged vessels (Hartnett *et al*, 2008).

Expression of different VEGF-A isoforms is also important in determining neovascular structure. The major VEGF-A isoforms (human 189, 165 and 121, all one amino acid shorter in mice) are generated by the alternative splicing of a single gene (Ferrara and Keyt, 1997). These isoforms generally exhibit comparable growth factor and permeability-enhancing activities. All bind the major VEGF-A receptors (VEGFR-2/Flk and VEGFR-1/Flt), and all except VEGF-A^120/1^ bind to a third receptor, neuropilin. However, several VEGF-A isoforms differ strikingly in their capacities to bind heparin, and cell surface and matrix heparans (Ferrara and Keyt, 1997). VEGF-A^120/1^ lacks heparan-binding sites altogether and diffuses freely in tissues. VEGF-A^188/9^ binds strongly to cell- and matrix-associated heparans and, at least until some threshold is exceeded, is confined locally. VEGF-A^164/5^ has intermediate heparin-binding, and therefore diffusion, properties.

These differences in the cell and matrix-binding capacities of the different VEGF-A isoforms have important consequences for vascular patterning. Mice engineered to express only VEGF-A^120^ develop to term but generate a capillary network with larger-sized vessels and fewer branch points (Ruhrberg *et al*, 2002). Similarly, tumour cells expressing only VEGF-A^120^ form small tumours with numerous, large peripheral blood vessels (presumably MV), but with few, small intratumour vessels (Grunstein *et al*, 2000; Tozer *et al*, 2008). Similar large, MV-like vessels can be generated by still smaller VEGF-As that result from matrix metalloprotease cleavage (Lee *et al*, 2005). The abnormal vascular pattern induced by VEGF-A^120^ has been attributed to its inability to bind to matrix and, therefore, to form the steep VEGF-A gradients that are thought to be necessary for generating normal blood vessels. Recent work has shown that growing capillaries are comprised of two types of endothelial cells: non-dividing ‘tip’ cells at the leading edge that extend filopodia and sprouts in response to VEGF-A gradients and ‘stalk’ cells that follow behind, divide and exhibit reduced filopodia and sprouting because of signalling through the Notch/delta 4 axis (Hellstrom *et al*, 2007). Thus, the shallow gradient generated by VEGF-A^120^ is thought to reduce the polarity of tip cells and to increase their proliferation rate, causing them to form large vessels with reduced branching.

In contrast, mice expressing only VEGF-A^188^ are reported to develop high vascular density and disorganized patterning with lining endothelial cells that exhibit increased, multidirectional filopodia (Ruhrberg *et al*, 2002). Tumour cells expressing only VEGF-A^188^ formed small tumours with numerous intratumour blood vessels and few large peripheral vessels (Grunstein *et al*, 2000). This pattern is understandable, in that VEGF-A^188^ is largely cell-bound and, therefore, can induce new blood vessels only in its immediate vicinity. Recently, fibrosarcoma cells overexpressing VEGF-A^188^ alone were found to generate better differentiated, narrower and pericyte-coated vessels with reduced hyperpermeability and greater resistance to vascular disrupting agents (Tozer *et al*, 2008).

Taken together, VEGF-A^120^ can be regarded as a long-range chemoattractant, involved in the initiation of vascular patterning, whereas the longer VEGF-As are critical for establishing the intricate, fine-patterning vessel architecture found in normal tissues. Matrix-bound and nontethered VEGF(s) clearly ‘provide different signalling outcomes, even though they act through the same cell surface receptor (VEGFR2)’ (Lee *et al*, 2005). In contrast, VEGF-A^164/5^, the isoform most commonly expressed by tumour cells, shares properties of both the 120/1 and 188/9 isoforms. Tumour cells overexpressing VEGF-A^164^ form large tumour masses with both abundant peripheral and internal blood vessels, that is, a vasculature typical of human tumours as well as of that generated in normal mouse tissues by Ad-VEGF-A^164^.

Finally, tortuosity is a property frequently exhibited by MV, VM, feeder arteries and draining veins. Tortuosity has generally been attributed to a restriction on lengthening, which is imposed by vessel anchoring at fixed points upstream and downstream. As a result, growing tumour vessels cannot extend linearly and so they coil.

## Why might an understanding of tumour blood vessel diversity be important clinically?

Agents that neutralise VEGF (e.g., Avastin, VEGF-TRAP) or its receptors have provided proof of principle that anti-angiogenesis can be a useful adjunct to tumour therapy (Jain *et al*, 2006). However, these approaches have not been curative and are not likely to be so for at least two reasons. First, they exert their effect only within a narrow therapeutic window in which their inhibitory effects on angiogenesis do not cause excessive toxicity; it has become apparent in recent years that normal blood vessels, and also the central nervous system, require VEGF-A for maintenance and are compromised when circulating VEGF-A falls below threshold levels (Carmeliet, 2003). Second, it is likely that approaches targeting VEGF/VEGFR damage only a subset of tumour blood vessels. MV and GMP are likely to be susceptible, but daughter blood vessels such as capillaries and VMs are not, nor are the feeder arteries and draining veins that result not from angiogenesis but from arteriogenesis and venogenesis. Unlike MV and GMP, these last vessels have acquired a complete and often multilayered pericyte or smooth muscle-cell coat, and lose detectable VEGFR-2 expression (unpublished data). As a result, they have lost dependency on exogenous VEGF-A and can survive indefinitely in a low VEGF-A environment. It is not surprising, therefore, that anti-VEGF/VEGFR approaches have effectively treated mouse tumours at early stages of their development, but have proved to be less successful in treating established mouse tumours that contain large numbers of more mature, smooth muscle-cell-coated blood vessels (Bergers *et al*, 2003). The same reasoning is likely to apply to human cancers. Human tumours are typically present for months or years before they are detected; as a result, MV and GMP are likely to account for only a small fraction of the total tumour vasculature.

## Conclusions

Tumours induce their vasculature through the unbalanced, local overexpression of a small number of growth factors, particularly VEGF-A. Tumour neovascularization thus differs significantly from the sprouting angiogenesis of normal vascular development, a process that is as yet not well understood, but that results, at least in part, from tightly regulated VEGF-A gradients and the balanced expression of many other growth factors and inhibitors. Tumours generate at least six distinct types of new blood vessels that differ from each other, and from the normal vasculature, with respect to organisation, structure and function. This classification has potential clinical and therapeutic significance. Current anti-VEGF/VEGFR approaches more likely target only a subset of angiogenic tumour blood vessels, that is, MV and GMP, that are dependent on exogenous VEGF-A. By contrast, the mature, smooth muscle-cell-coated daughter blood vessels, as well as the large feeder arteries and draining veins that result from arteriogenesis and venogenesis, are unlikely to be targeted, as they have lost VEGF-A dependence. Feeder arteries and draining veins would seem to be particularly valuable targets, because they supply and drain all of the smaller, angiogenic vessels enclosed within the tumour mass. Finding drugable targets on these blood vessels will require them to be better characterized. However, the genomic and proteomic revolution should make it possible to find such targets, especially because (as success with anti-VEGF/VEGFR drugs has shown) differences between tumour and normal blood vessels need not be absolute for therapy to be effective.

## Figures and Tables

**Table 1:** Classification of tumour blood vessels^a^

MV	Greatly enlarged, tortuous, thin-walled, pericyte-poor hyperpermeable sinusoids
Capillaries	Similar to normal capillaries
GMP	Tangles of tiny vessels immersed in a complex mixture of irregularly ordered pericytes and extensive multilayered basement membrane
VM	Large vessels with an irregular coat of smooth muscle cells
FA and DV	Greatly enlarged, tortuous smooth muscle-cell-coated vessels that supply and drain the complex of angiogenic blood vessels

DV=draining veins; FA=feeder arteries; GMP=glomeruloid microvascular proliferations; MV=mother vessels; VM=vascular malformations.

MV, capillaries, GMP and VM result from angiogenesis; FA and DV result, respectively, from arteriogenesis and venogenesis.

a In addition, red blood-cell-filled spaces lined by tumour cells rather than by vascular endothelium have been described in some tumours, particularly ocular melanomas, and are referred to as ‘vascular mimicry’ (Folberg *et al*, 2000).
